# Coronary Artery Calcium Score Improves Risk Assessment of Symptomatic Patients in Low-Risk Group Based on Current Guidelines

**DOI:** 10.31083/j.rcm2406162

**Published:** 2023-06-06

**Authors:** Chengjian Wang, Xiaomeng Zhang, Chang Liu, Chao Zhang, Guolei Sun, Jia Zhou

**Affiliations:** ^1^Department of Cardiology, Tianjin Chest Hospital, 300222 Tianjin, China; ^2^Department of Emergency, Tianjin University Jinnan Hospital, 300350 Tianjin, China; ^3^Thoracic Clinical College, Tianjin Medical University, 300070 Tianjin, China

**Keywords:** coronary artery disease, stable chest pain, pretest probability, coronary artery calcium score, coronary computed tomography angiography, risk assessment

## Abstract

**Background:**

The guidelines for evaluation and diagnosis of stable chest pain (SCP) released by American societies in 2021 (2021 GL) and European Society of Cardiology (ESC) in 2019 both recommended the estimation of pretest probability (PTP) by ESC-PTP model. Further risk assessment for the low-risk group according to 2021 GL (ESC-PTP ≤15%) is important but still remains unclear. Thus, the present study intended to comprehensively investigate the diagnostic and prognostic value of coronary artery calcium score (CACS) in these low-risk patients.

**Methods:**

From January 2017 to June 2019, we initially enrolled 8265 patients who were referred for CACS and coronary computed tomography angiography (CCTA) for the assessment of SCP. PTP of each patient was estimated by ESC-PTP model. Patients with ESC-PTP ≤15% were finally included and followed up for major adverse cardiovascular event (MACE) and utilization of invasive procedures until June 2022. The degree of coronary artery disease (CAD) on CCTA was defined as no CAD (0%), nonobstructive CAD (1–49%) and obstructive CAD (≥50%). Multivariate Cox proportional hazards and Logistic regression models were used to calculate adjusted hazard ratios (HRs) and odds ratios (ORs) with 95% confidence intervals (CIs), respectively.

**Results:**

A total of 5183 patients with ESC-PTP ≤15% were identified and 1.6% experienced MACE during the 4-year follow-up. The prevalence of no CAD and obstructive CAD decreased and increased significantly (*p <* 0.0001) in patients with higher CACS, respectively, and 62% had nonobstructive CAD among those with CACS >0, resulting in dramatically increasing ORs for any stenosis ≥50% and >0% across CACS strata. Higher CACS was also associated with an elevated risk of MACE (adjusted HR of 3.59, 13.47 and 6.58 when comparing CACS = 0–100, CACS >100 and CACS >0 to CACS = 0, respectively) and intensive utilization of invasive procedures.

**Conclusions:**

In patients for whom subsequent testing should be deferred according to 2021 GL, high CACS conveyed a significant probability of substantial stenoses and clinical endpoints, respectively. These findings support the potential role of CACS as a further risk assessment tool to improve clinical management in these low-risk patients.

## 1. Introduction 

Current international guidelines for the evaluation and diagnosis of patients with stable chest pain (SCP) suspected of chronic coronary syndrome (CCS) recommended pretest probability (PTP) stratification before cardiac imaging testing (CIT), such as coronary computed tomographic angiography (CCTA) [1, 2]. This is true whether this be the guideline released by European Society of Cardiology (ESC) in 2019 [1] or American societies in 2021 (2021 GL) [2]. For the estimation of PTP, both guidelines adopted the ESC-PTP model based on age, sex and symptoms [3]. Although ESC-PTP model has been externally validated in different CCTA-based cohorts of SCP patients, the details were inconclusive for determination of the low-risk group in which further CIT should be deferred for patients [4, 5, 6, 7, 8, 9]. A recent study demonstrated that the impact of implementing 2021 GL, which assigned all patients with ESC-PTP ≤15% to the low-risk group, remained to be elucidated for the modest improvement in efficiency and outcomes [7]. This concern is particularly crucial, because patients with ESC-PTP ≤15% account for approximately more than half of the current SCP cohorts, and these patients may benefit from optimal medical therapy (OMT) and potentially revascularization, despite low rates of obstructive coronary artery disease (CAD) and major adverse cardiovascular event (MACE) [4, 5, 6, 7, 8, 9, 10, 11]. Consequently, further risk assessment for patients in the the low-risk group according to 2021 GL is warranted.

To address this issue, 2021 GL recognized coronary artery calcium score (CACS), a direct marker of calcified coronary atherosclerosis, as a quick, lower-radiation and relatively inexpensive tool for further risk assessment [2]. For patients with SCP and no known CAD categorized as low-risk, the 2021 GL adopted a Class 2A recommendation regarding CACS as a reasonable first-line test for excluding calcified plaque and identifying patients with a low likelihood of obstructive CAD [2]. This recommendation was supported by a meta-analysis of 79,903 patients with SCP which found the association between CACS = 0 and the low prevalence of CAD and MACE [12] and a cohort study of 33,552 patients without obstructive CAD which demonstrated that the absolute benefit of directly proportional with the CAD burden measured by CACS [13]. Our previous research from the CCTA Improves Clinical Management of Stable Chest Pain (CICM-SCP) registry also confirmed the strong potential of CACS to improve effectiveness of the diagnostic strategy [10, 11]. However, the clinical value of CACS for patients in the low-risk group according to 2021 GL still remains unclear. A recent study demonstrated a 5-year warranty period for a CACS of 0 in low-risk population [14]. Thus, the present study aims to comprehensively investigate the diagnostic and prognostic impact of CACS, as well as the association between CACS and subsequent utilization of invasive procedures, in these low-risk patients (ESC-PTP ≤15%).

## 2. Materials and Methods

### 2.1 Study Population

**Fig. 1.**
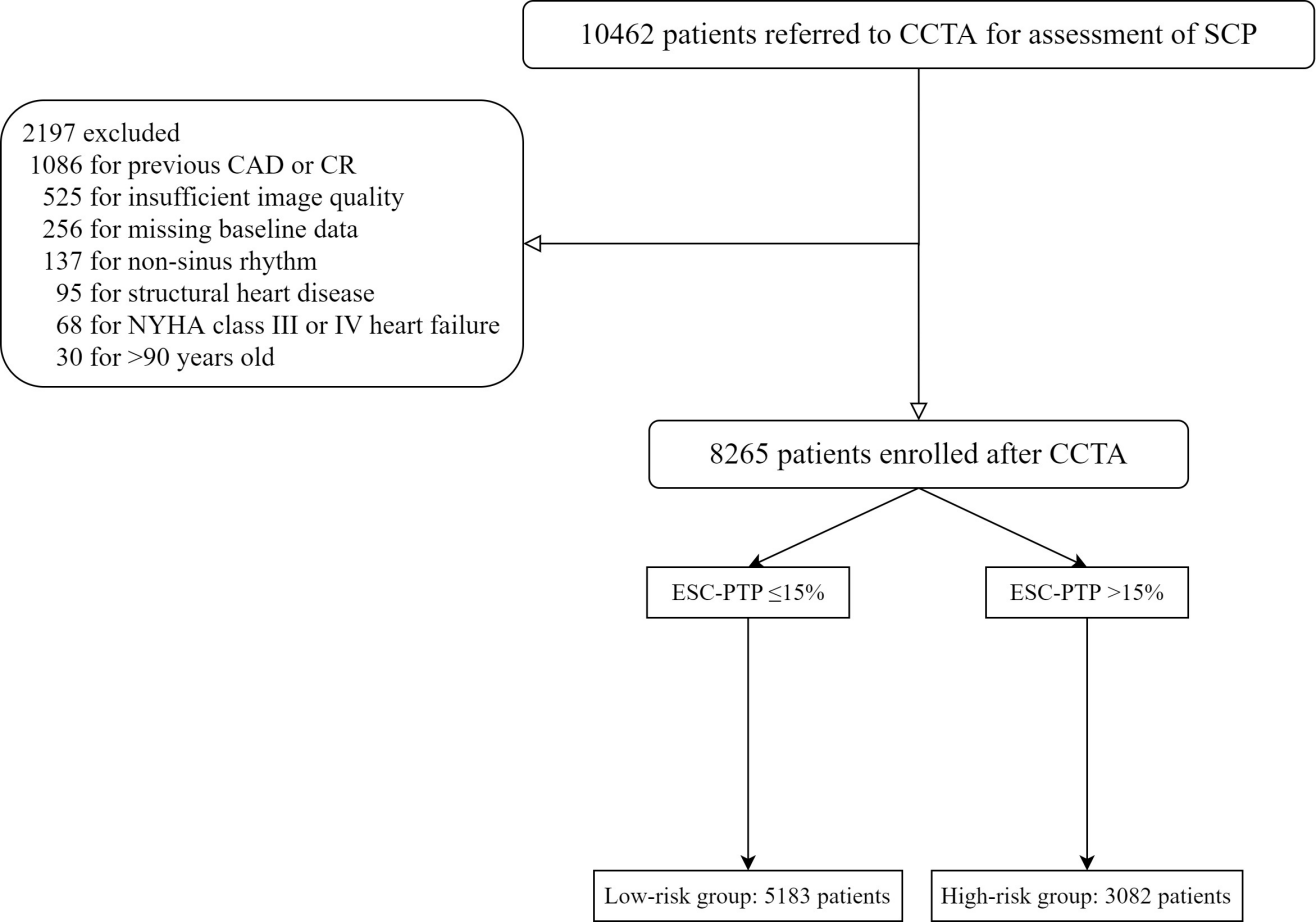
**Study flowchart**. SCP, stable chest pain; CCTA, coronary computed tomographic angiography; CAD, coronary artery disease; PTP, pretest probability; ESC, European Society of Cardiology; CR, coronary revascularization; NYHA, New York Heart Association.

Briefly, the CICM-SCP registry is a prospective and ongoing cohort of patients who were referred to CCTA as first-line CIT for the assessment of SCP suspected of CCS (ClinicalTrials.gov Identifier: NCT04691037). Details about the registry have been previously described [10, 11]. As shown in Fig. 1, from January 2017 to June 2019, 8265 patients were finally enrolled in the present study. The present study was conducted in accordance with the Declaration of Helsinki and approved by the Ethics Committees of local institutions. All participants gave informed consent.

### 2.2 Baseline Clinical Characteristics and Risk Assessment According to 2021 GL

Baseline clinical data were prospectively collected and defined as described previously [10, 11]. ESC-PTP for each patient was estimated using age, sex and symptoms [3]. According to the recommendations of 2021 GL, CIT should be deferred for a patient with ESC-PTP ≤15% and referred to a patient with ESC-PTP >15%. Thus, we classified patients with ESC-PTP ≤15% into low-risk group and the present study mainly focused on them.

### 2.3 CACS and CCTA

The image scanning and result interpretation of CACS and CCTA were conducted as described previously [10, 11]. Based on the results of previous studies, CACS was categorized into 3 groups: 0, 0–100 and >100 [12, 13]. CACS = 100–400 and >400 were merged into one group for the relatively small number of patients. Each coronary segment with >2 mm diameter was analyzed in the presence of coronary diameter stenosis. According to the updared Coronary Artery Disease–Reporting and Data System [15], the maximal degree of coronary diameter stenosis was defined as no CAD (0%), nonobstructive CAD (1–49%) and obstructive CAD (≥50%).

### 2.4 Follow Up and Study Endpoints

The details about definition of study endpoint and follow-up information collection were described previously [10, 11]. After CCTA, all patients were followed until June 2022. The primary endpoint was MACE, defined as a composite of all-cause death and nonfatal myocardial infarction (MI). All-cause death was used rather than cardiovascular death to eliminate the need for possibly difficult adjudication of causes of death, especially given the relatively low mortality. The secondary endpoint included invasive coronary angiography (ICA) utilization and referral to revascularization, including percutaneous coronary intervention (PCI) and coronary artery bypass graft (CABG). For a patient suffering repeat endpoints, we mainly focused on the first one [16].

### 2.5 Statistical Analysis

All statistical analyses were performed using R (version 4.0.3; R Foundation for Statistical Computing, Vienna, Austria) or MedCalc (version 15.2.2, MedCalc Software, Mariakerke, Belgium). Two-tailed *p <* 0.05 was considered statistically significant. Student *t*-test was used to compare normally distributed continuous data, and Mann-Whitney U-test was used to compare nonnormally distributed continuous data. Categorical variables were compared using χ^2^ test or Fisher exact test as appropriate. We constructed Kaplan–Meier curves for cumulative event-free estimates survival from the first of the following: endpoints of concern, death, the end of follow up or loss to follow up. Cox proportional hazard regressions were used to calculate adjusted hazard ratios (HRs) and 95% confidence intervals (CIs), which assessed CACS to the time to first MACE (or censoring). The proportional hazard assumption was assessed using Schoenfeld residuals and was met for all models. Logistic regression models were used to calculate adjusted odds ratios (OR) and 95% CI which evaluate independent relationships between CACS and CAD or utilization of invasive procedures. These multivariate models were all adjusted for age, sex, hypertension, hyperlipidemia, diabetes, smoking, family history of CAD and symptoms.

## 3. Results 

### 3.1 Study Population and Baseline Characteristics 

Table 1 shows baseline clinical characteristics of the study cohort by low- and high-risk group according to 2021 GL. The mean age was 65 years, with a standard deviation of 8.2 years and the median CACS was 4 (interquartile range: 0–84). Of the 8265 patients, 52% were men, 59% had angina pectoris, and 58% had a CACS of 0. Except family history of CAD, there were significant differences between low and high-risk group. Furthermore, as shown in Table 2, there were significant differences in all baselines clinical characteristics using 2 CACS cut-points (CACS >0 and CACS = 0; CACS >100, CACS = 0–100 and CACS = 0) among the 5183 patients in low-risk group according to 2021 GL.

**Table 1.** **Baseline characteristics of patients by low and high risk group**.

Characteristic		Total	Low-risk group	High-risk group	*p*
		(n = 8265)	(n = 5183)	(n = 3082)	
${\mathrm{Age}}^{a}$		56.75 ± 8.24	50.98 ± 8.63	66.45 ± 9.27	<0.0001
Male		4298 (52)	1866 (36)	2432 (79)	<0.0001
Diabetes		992 (12)	363 (7)	629 (20)	<0.0001
Hypertension		3306 (40)	1918 (37)	1388 (45)	<0.0001
Hyperlipidemia		3058 (37)	1607 (31)	1451 (47)	<0.0001
Smoking		2314 (28)	1347 (26)	967 (31)	0.0003
Family history of CAD		2066 (25)	1280 (25)	786 (26)	0.4278
Symptoms					<0.0001
	Nonanginal	3388 (41)	2954 (57)	434 (14)	
	Atypical anginal	3141 (38)	1814 (35)	1327 (43)	
	Typical anginal	1736 (21)	415 (8)	1321 (43)	
${\mathrm{CACS}}^{b}$		4 (0–84)	0 (0–72)	31 (0–268)	<0.0001

CACS, coronary artery calcium score; CAD, coronary artery disease. Values are presented as n (%) unless stated otherwise. ^a^ years, mean ± standard deviation. ^b^ median (25th–75th).

**Table 2.** **Baseline Characteristics by CACS in low-risk group**.

Characteristic		CACS					
		0	>0	* $p^{b}$ *	0–100	>100	* $p^{c}$ *
		(n = 3006)	(n = 2177)		(n = 1296)	(n = 881)	
${\mathrm{Age}}^{a}$		47.62 ± 9.27	55.62 ± 10.16	<0.0001	52.96 ± 10.58	59.53 ± 11.07	<0.0001
Male		962 (32)	904 (42)	<0.0001	505 (39)	399 (45)	<0.0001
Diabetes		90 (3)	273 (13)	<0.0001	117 (9)	156 (18)	<0.0001
Hypertension		1052 (35)	866 (40)	0.0005	493 (38)	373 (42)	0.0003
Hyperlipidemia		812 (27)	795 (37)	<0.0001	428 (33)	367 (42)	<0.0001
Smoking		631 (21)	716 (33)	<0.0001	363 (28)	353 (40)	<0.0001
Family history of CAD		721 (24)	574 (26)	<0.0001	324 (25)	250 (28)	0.0112
Symptoms				<0.0001			<0.0001
	Nonanginal anginal	1833 (61)	1121 (51)		713 (55)	408 (46)	
	Atypical anginal	992 (33)	82 (38)		467 (36)	355 (40)	
	Typical anginal	181 (6)	234 (11)		116 (9)	118 (14)	

CACS, coronary artery calcium score; CAD, coronary artery disease. Values are presented as n (%) unless stated otherwise. ^a^ years, mean ± standard deviation. ^b^ *p* value for comparison of CACS = 0 and CACS >0. ^c^ *p* value for comparison of CACS = 0, 0–100 and >100.

### 3.2 CAD on CCTA 

**Fig. 2.**
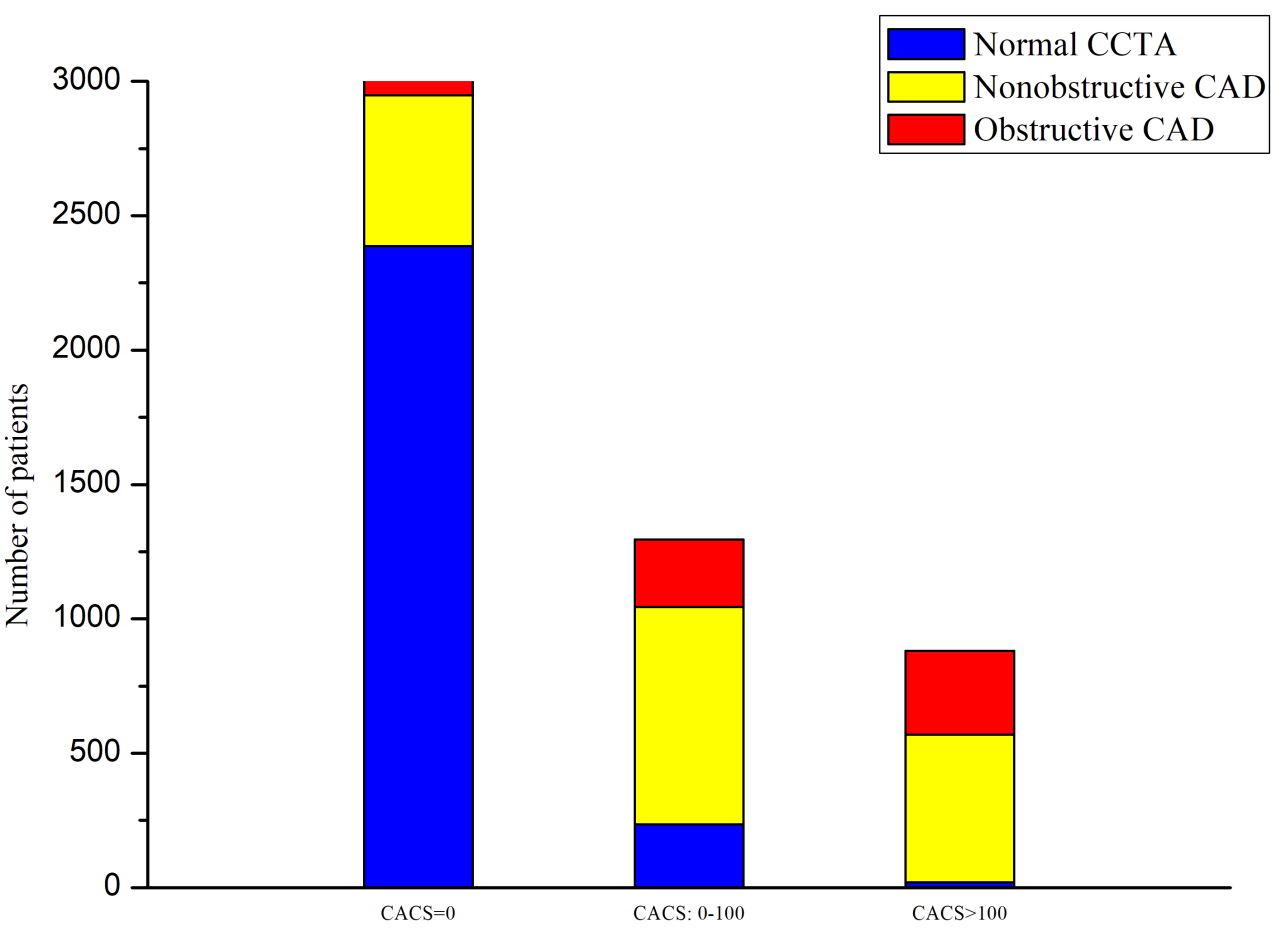
**Distribution of obstructive, nonobstructive and no CAD on CCTA according to CACS = 0, 0–100 and >100**. CCTA, coronary computed tomographic angiography; CACS, coronary artery calcium score; CAD, coronary artery disease.

The associations between CACS and CAD on CCTA are manifested in Fig. 2. Overall, obstructive, nonobstructive, and no CAD was identified on CCTA in 622 (12%), 1918 (37%) and 2643 (51%) patients, respectively. The prevalence of no CAD and obstructive CAD decreased and increased significantly (*p <* 0.0001) in patients with higher CACS, respectively. Among those with CACS = 0, most (79%, 2387/3006) had no CAD whereas only less than 2% (58/3006) had obstructive CAD. Conversely, more than 19% (252/1296) and 35% (312/881) had obstructive CAD among those with CACS = 0–100 and CACS >100, respectively.

As shown in Table 3, the adjusted ORs for any stenosis ≥50% increased stepwise with higher CACS. It was worth noting that more than half (62%, 808/1296 and 62%, 549/881) had nonobstructive CAD among those with CACS = 0–100 and CACS >100, respectively. Thus, the multivariable ORs for any stenosis >0% followed the same pattern, with more dramatically increasing across CACS strata (Table 3). Additionally, patients with CACS >0 compared with those with CACS = 0 had 7.49 (95% CI: 2.85–12.63) and 19.71 (95% CI: 10.85–29.47) higher odds of having obstructive CAD and any CAD, respectively (Table 3).

**Table 3.** **The estimated risks of different endpoints according to CACS**.

CACS groups	CAD on ${\mathrm{CCTA}}^{a}$		Invasive ${\mathrm{procedure}}^{a}$		${\mathrm{MACE}}^{a}$
	Any stenosis >0%	Any stenosis ≥50%	ICA	Revascularization	
CACS = 0	Reference	Reference	Reference	Reference	Reference
CACS = 0–100	10.49	8.15	8.37	9.52	3.59
	(4.62 to 17.05)	(4.27 to 13.62)	(4.02 to 15.39)	(2.37 to 22.84)	(1.17 to 6.23)
CACS >100	83.06	21.74	25.91	32.69	13.47
	(21.65 to 150.37)	(9.38 to 35.01)	(10.35 to 51.93)	(10.85 to 74.13)	(4.29 to 28.15)
CACS = 0	Reference	Reference	Reference	Reference	Reference
CACS >0	19.71	7.49	14.38	18.34	6.58
	(10.85 to 29.47)	(2.85 to 12.63)	(6.25 to 27.41)	(5.96 to 39.72)	(2.07 to 15.39)

MACE, major adverse cardiovascular event; CACS, coronary artery calcium score; CAD, coronary artery disease; CCTA, coronary computed tomography angiography; ICA, invasive coronary angiography. ^a^ The adjusted odds ratios or hazard ratios with 95% confidence interval for CACS were estimated by multivariate regression models accounting for baseline characteristics.

### 3.3 Invasive Procedures 

**Fig. 3.**
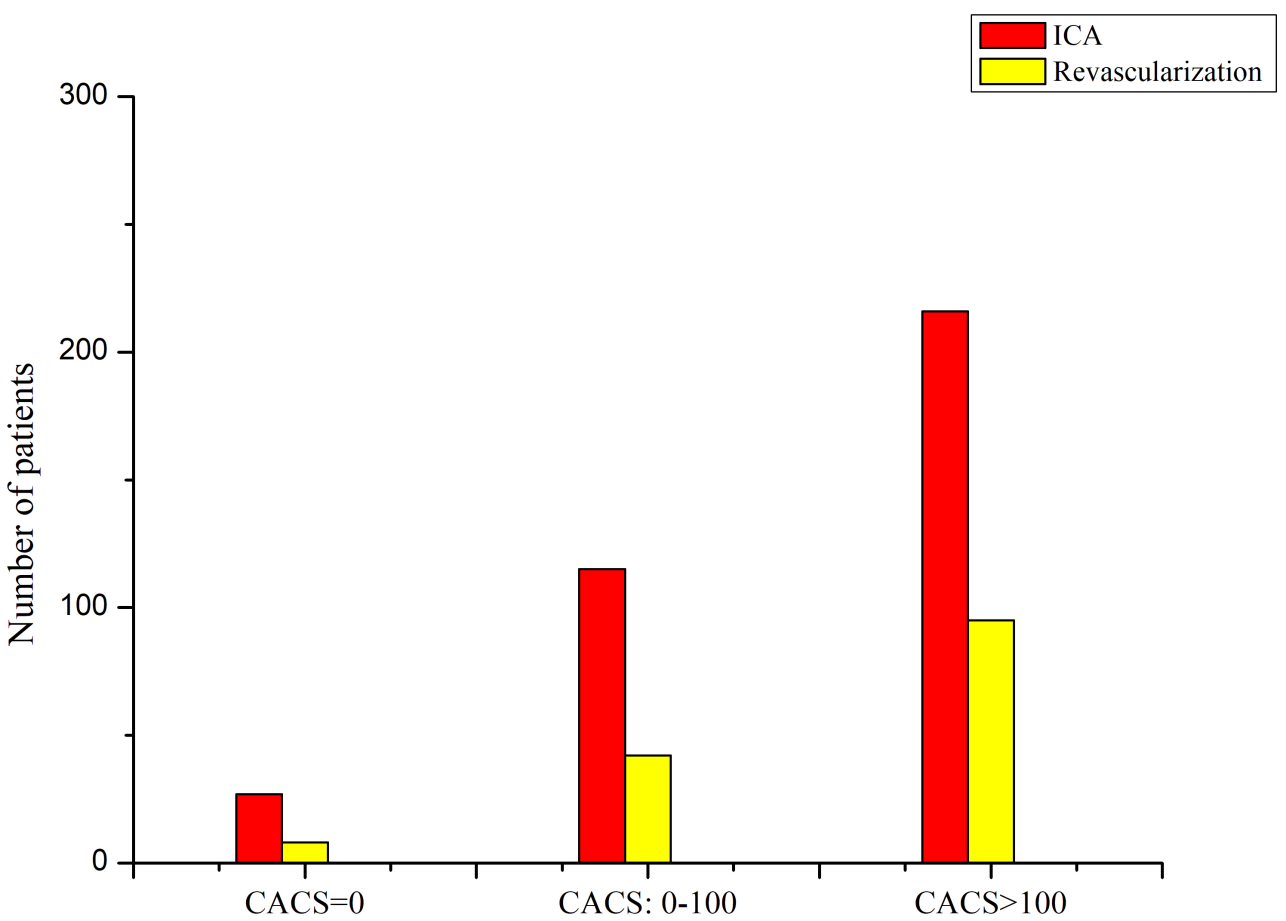
**Utilization of invasive procedures after CCTA according to CACS = 0, 0–100 and >100**. ICA, invasive coronary angiography; CACS, coronary artery calcification score; CCTA, coronary computed tomographic angiography.

Fig. 3 illustrated the utilization of ICA and revascularization according to CACS. After CCTA, a total of 358 and 145 patients had at least one ICA and revascularization (118 PCI and 27 CABG), respectively. The utilization of ICA and revascularization increased steadily (*p <* 0.0001) in patients with higher CACS, respectively. Among those with CACS = 0, less than 0.9% (27/3006) and 0.3% (8/3006) had ICA and revascularization, respectively. The proportions were significantly (*p <* 0.0001) elevated to 9% (115/1296) and 3% (42/1296) in patients with CACS = 0–100 and 25% (216/881) and 11% (95/881) in patients with CACS >100, respectively. As a result, there was a graded increase in the adjusted ORs of ICA and revascularization with the degree of CACS present, respectively (Table 3). Compared with CACS = 0 as the reference, patients with CACS >0 were more likely to receive ICA (OR: 14.38, 95% CI: 6.25 to 27.41) and revascularization (OR: 18.34, 95% CI: 5.96 to 39.72) after CCTA, respectively.

### 3.4 MACE

**Fig. 4.**
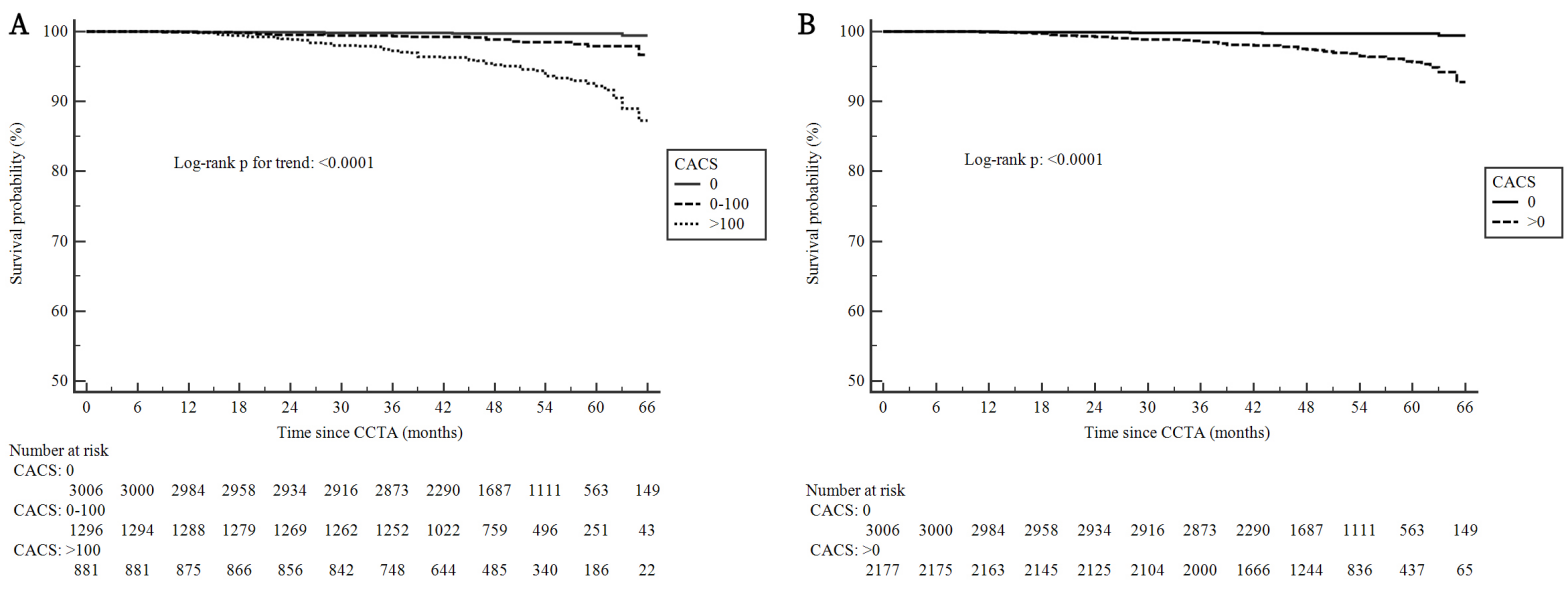
**Kaplan–Meier curves of patients surviving free from the first MACE after CCTA according to CACS**. (A) CACS = 0, 0–100 and >100. (B) CACS = 0 and >0. MACE, major adverse cardiovascular event; CACS, coronary artery calcification score; CCTA, coronary computed tomographic angiography.

Patients were followed up for a median of 49 (interquartile range: 41 to 57) months and 382 (7%) were lost to follow-up. During the 4-year follow-up, 1.6% (83/5183) among low-risk patients experienced MACE: 15 patients died and 68 patients suffered from nonfatal MI. The corresponding number among high-risk patients was 4.4% (136/3082), 28 and 108, respectively. In low-risk group, the number of MACE for patients with a CACS of 0 and >0 was 10 and 73, respectively. The Kaplan–Meier curves demonstrated that both the discrepancies of cumulative rates among CACS = 0, 0–100 and >100 (Fig. 4A, Log-rank *p *for trend <0.0001) and between CACS = 0 and >0 (Fig. 4B, Log-rank *p <* 0.0001) appeared to be mainly attributed to the more frequent occurrences of MACE in the moderate and late stage of follow-up. These yielded an adjusted HR of 3.59 (95% CI: 1.17 to 6.23), 13.47 (95% CI: 4.29 to 28.15) and 6.58 (95% CI: 2.07 to 15.39) when comparing patients with CACS = 0–100, CACS >100 and CACS >0 to those with CACS = 0, respectively. Graphically, the warrant period of CACS = 0 in the present study was 5-year due to the extremely low frequency of MACE.

## 4. Discussion

In this CCTA-based and long-term follow-up cohort study, consecutive patients with SCP suspected of CCS were classified into low and high-risk group according to the recommendations of 2021 GL. Although a percentage of patients in the low-risk group had different degrees of CAD on CCTA or suffered clinical events, higher CACS was associated with an increased likelihood of CAD (especially nonobstructive CAD), intensive utilization of invasive procedures and elevated risk of MACE with stepwise grades (CACS = 0, 0–100 and CACS >100) or presence (CACS >0) v.s. absence (CACS = 0).

Several studies have shown a low diagnostic and prognostic yield of CIT in routine testing [17, 18, 19, 20]. Hence, the evaluation of SCP suggestive of CCS remains a challenge for physicians with significantly increased costs related to these patients [21, 22]. The 2021 GL recommended risk assessment by ESC-PTP model and for patients in low-risk group (ESC-PTP ≤15%), further CIT should be deferred [2]. Consistent with other studies [4, 5, 6, 7, 8, 9, 10, 11], the present study demonstrated that although the low-risk group had less risk burden and MACE than the high-risk group did, there was still a considerable proportion of patients in the low-risk group had obstructive CAD detected by CCTA. An increasing body of evidences has pointed to the fact that in the SCP population, approximately one-third of patients with CACS >0 had obstructive CAD [12], which was supported by the substantially high odds of having obstructive CAD for patients with CACS >0, especially >100, after controlling for confounders by a large range of prognostic variables for this study.

Interestingly, more than 60% patients had nonobstructive CAD among those with CACS >0, leading to dramatically increased OR of having any CAD across CACS strata. Recent literature suggests that most MACE occurred in patients with nonobstructive CAD detected by CCTA [23, 24]. In a large-scale trial, CCTA arm demonstrated a lower rate of MACE than the traditional care arm did during a long follow-up of 5 years, which was mainly attributed to greater intensity of OMT in response to visualize (mostly nonobstructive) CAD [25]. A recent study of 33,552 consecutive patients without obstructive CAD determined by CCTA found statin therapy post-CCTA was associated to a risk reduction of MACE in 5-year follow-up, with the number need to treat of 36, 24 and 13 in patients with CACS = 0–100, 100–400 and >400, respectively [13]. CACS may have the potential to provide the opportunity for the screening of subclinical atherosclerosis to improve preventive OMT. Despite the intensive utilization of invasive procedures associated with higher CACS, most MACE arose in the moderate and late stages of follow-up on Kaplan-Meyer curves, emphasizing the important role of OMT in low-risk patients without CACS = 0.

In terms of the clinical practice, not performing any CIT is a difficult concept to embrace even in the low-risk group according to 2021 GL [26]. Thus, the 2021 GL offered the option to pursue CACS as a quick, lower-radiation and relatively inexpensive tool for further risk assessment in the low-risk group, but only at a strength of recommendation with 2a and a level of evidence with B provides little guidance on the use of CACS [2]. This is the first CCTA-based and longitudinal study comprehensively investigating the clinical value of CACS in a real-world cohort of patients assigned to low-risk group by 2021 GL, leading to a potential CACS-based paradigm for specific risk assessment in these patients. For those with CACS >0, OMT based on recent primary and secondary prevention guidelines should be referred [1, 27, 28, 29]. For those with CACS >100, additional CIT, such as CCTA, may be considered. However, as shown in the present study and other research, CACS may perform worse in some subgroups [30, 31, 32], CACS of 0 is not a complete guarantee to de-escalate or defer subsequent OMT and necessary CIT.

Several other limitations of the present study merit discussion. First, this was a subgroup analysis of an observational and natural history registry. Indications for CCTA and post–CCTA management relied on the decision making of local physicians in a nonrandomized fashion. Follow-up data indicating favorable outcomes were derived from patients whose clinical care benefited from guidance by CCTA. The influence of potential selection bias could not be completely excluded, although we used multivariable adjustment to control for potential confounding by a large range. Second, our previous studies [10, 11], as well as other similar studies [4, 30, 31, 32, 33] have demonstrated that applying a CACS-based estimation of PTP to all SCP patients seemed to have been more potential to effectively identify patients with low-risk. Thus, multicentric and multiethnic randomized controlled trials are needed to assess whether incorporation of CACS as a gatekeeper in the low-risk group according to 2021 GL is noninferior to current safety and could lead to meaningful reductions in downstream CIT and health care expenditure. Third, CAD was documented using CCTA in this study. Previous studies have demonstrated that CCTA had a high negative predictive value compared with ICA [34, 35]. Thus CCTA offered robust assurance to exclude obstructive CAD [36]. Fourth, our study did not include patients with dyspnea, and the conclusions should not be extrapolated to patients with known CAD, acute chest pain, no chest pain or classified into high-risk group according to 2021 GL [37].

## 5. Conclusions

This is the first CCTA-based study to investigate the diagnostic and long-term prognostic value of CACS. As well we investigated the association between CACS and subsequent utilization of invasive procedures, in patients with SCP suspected of CCS and assigned to the low-risk group according to 2021 GL. Although there is still a percentage of these low-risk patients having different degrees of CAD on CCTA or suffering MACE, high CACS conveyed a significant probability of substantial stenoses and clinical endpoints, respectively. These findings support the potential role of CACS as a further risk assessment tool to improve clinical management in patients for whom subsequent CITs have been deferred based on recommendations of 2021 GL.

## Data Availability

The datasets used and/or analyzed during the current study are available from the corresponding author on reasonable request.
